# Abstainers and Life Insurance

**Published:** 1889-04-20

**Authors:** Edward T. Clifford

**Affiliations:** Scottish Metropolitan Life Assurance Company, 8, King-street, Cheapside


### Editor's Letter-Box.

[Our Correspondents are reminded that prolixity is a great bar to publication, and that brevity of style and conciseness of statement greatly facilitate early insertion.]

### ABSTAINERS AND LIFE ASSURANCE.

I read the communication which may be found on page 15 of last week's *Hospital*. The statement requires considerable qualifications, and I hope you will agree with me that in its present state it is unjust to other insurance companies, and, indeed, other assurers.

The United Kingdom Temperance and General has always obtained tremendous advertisements (chiefly gratuitous) by comparison being instituted between their abstainers' and ordinary sections, but I believe I am justified in saying that the method they adopt entirely destroys a just comparison. It should be understood that if a man joining under the temperance section becomes a backslider, he is immediately put into the ordinary section, and participates in that class's bonus. Now, it is notorious that a man who breaks the pledge under such circumstances is not likely to be so good a life as a moderate drinker, and it is obviously unfair to the "moderates" that their chance of bonus should be destroyed simply to benefit the abstainers.

The method one society recently adopted was that if an abstainer became a backslider he was put into a class by himself, and debarred from all future bonuses, and this appears to me to be a more equitable arrangement than that adopted by the United Kingdom Temperance and General.

I do not desire to say anything that will cast a slur on the office in question or upon temperance principles, for I recognise the immense amount of good the temperance cause is doing. At the same time, I am not satisfied that it has been clearly demonstrated by unbiassed evidence that the moderate drinker is inferior in length of days to the total abstainer.

Edward T. Clifford.

Scottish Metropolitan Life Assurance Company,
8, King-street, Cheapside, April 11, 1889.

